# Safety and efficacy of ombitasvir – 450/r and dasabuvir and ribavirin in HCV/HIV-1 co-infected patients receiving atazanavir or raltegravir ART regimens

**DOI:** 10.7448/IAS.17.4.19500

**Published:** 2014-11-02

**Authors:** Joseph J Eron, Jay Lalezari, Jihad Slim, Joseph Gathe, Peter J Ruane, Chia Wang, Richard Elion, Gary Blick, Amit Khatri, Yiran B Hu, Krystal Gibbons, Linda Fredrick, Melannie Co, Ronald D'Amico, Barbara Da Silva-Tillmann, Roger Trinh, Mark S Sulkowski

**Affiliations:** 1 Division of Infectious Diseases, University of North Carolina, Chapel Hill, NC, USA; 2 Quest Clinical Research, San Francisco, CA, USA; 3 Infectious Disease Medicine, St. Michael's Medical Center, Newark, NJ, USA; 4 Infectious Disease, Cure C. Consortium, Houston, TX, USA; 5 Peter J. Ruane, MD, Inc., Infectious Disease, Los Angeles, CA, USA; 6 Infectious Diseases, Virginia Mason Medical Center, Seattle, WA, USA; 7 Clinical Medicine, Whitman-Walker Health, Washington, DC, USA; 8 Infectious Diseases, CIRCLE CARE Center, Norwalk, CT, USA; 9 Clinical Pharmacokinetics, AbbVie Inc., North Chicago, IL, USA; 10 Statistics, AbbVie Inc., North Chicago, IL, USA; 11 Clinical Research Management, AbbVie Inc., North Chicago, IL, USA; 12 Medical Review, AbbVie Inc., North Chicago, IL, USA; 13 Medical Affairs, AbbVie Inc., North Chicago, IL, USA; 14 Medical Safety Evaluation, AbbVie Inc., North Chicago, IL, USA; 15 Antiviral Global Project Team, AbbVie Inc., North Chicago, USA; 16 Viral Hepatitis Center, Johns Hopkins University, Baltimore, MD, USA

## Abstract

**Objective:**

Whether concomitant HIV antiretroviral therapy (ART) affects the safety and efficacy of interferon-free HCV therapies or whether HCV treatment may negatively affect HIV control is unclear. We assessed the 3 direct-acting antiviral (3D) regimen of ombitasvir, ABT-450 (identified by AbbVie and Enanta; co-dosed with ritonavir) and dasabuvir with ribavirin (RBV) in HCV/HIV-1 co-infected patients with and without cirrhosis, including HCV treatment-experienced, receiving atazanavir (ATV)- or raltegravir (RAL)-based ART therapy.

**Methods:**

HCV genotype 1-positive treatment-naïve or pegIFN/RBV-experienced patients, with or without Child-Pugh A cirrhosis, CD4+ count ≥200 cells/mm^3^ or CD4 + % ≥14%, and plasma HIV-1 RNA suppressed on stable ART received open-label 3D + RBV for 12 or 24 weeks. Rates of HCV-sustained virologic response at post-treatment weeks 4 and 12 (SVR4 and SVR12, respectively) and bilirubin-related adverse events (AEs) are reported from post-hoc analyses for subgroups defined by treatment duration and ART regimen.

**Results:**

The SVR12 rate for patients receiving 12 weeks of 3D + RBV was 93.5% with comparable rates in patients receiving either ATV (93.8%) or RAL therapy (93.3%) (Table 1). The SVR4 rate for the 24-week arm was 96.9% with a single virologic breakthrough at treatment week 16 in a patient receiving RAL therapy. Patients receiving concomitant ATV had more AEs related to indirect hyperbilirubinemia including ocular icterus, jaundice and grade 3 or 4 elevations in total bilirubin (predominantly indirect). No patient discontinued the study due to AEs, and no serious AEs were reported during or after treatment. No patient had a confirmed plasma HIV-1 RNA value ≥200 copies/mL during the treatment period.

**Conclusions:**

In this first study to evaluate an IFN-free regimen in HCV genotype 1-positive treatment-naïve and experienced patients with HIV-1 co-infection, including those with cirrhosis, high rates of SVR were comparable to those with HCV monoinfection. Indirect hyperbilirubinemia was consistent with the known ABT-450 inhibition of the OATP1B1 bilirubin transporter, RBV-related haemolytic anaemia and inhibitory effect of ATV on bilirubin conjugation. The laboratory abnormalities and AEs observed did not negatively affect treatment response or lead to treatment discontinuation.

**Table 1:** Efficacy and safety of 12 or 24-week 3D+RBV in HCV/HIV coinfected patients subgrouped by HIV ART regimen

	12-week 3D + RBV	12-week 3D + RBV	24-week 3D + RBV	24-week 3D + RBV
Parameter, n/N (%)	ATV	RAL	ATV	RAL
SVR4	15/16 (93.8)	14/15 (93.3)	12/12 (100)	19/20 (95.0)
SVR12	15/16 (93.8)	14/15 (93.3)	NA	NA
**Adverse events and laboratory abnormalities** Ocular icterus Jaundice	5/16 (31.3) 2/16 (12.5)	0 0	1/12 (8.3) 0	0 0
Total bilirubin Grade 3 Grade 4	8/16 (50.0) 1/16 (6.3)	2/15 (13.3) 0	6/12 (50.0) 0	0 0
Aspartate aminotransferase Grade 3 Grade 4	0 0	0 0	0 0	1/20 (5.0) 0

